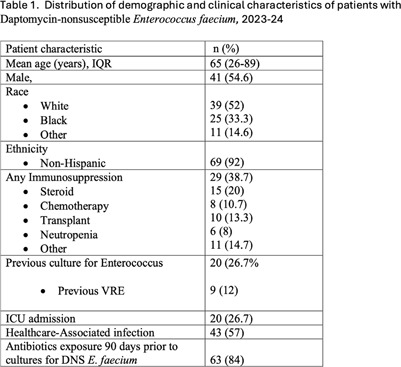# Clinical Characteristics and Antibiotic Exposure History in 75 Patients with Daptomycin-Nonsusceptible Enterococcus faecium

**DOI:** 10.1017/ash.2025.364

**Published:** 2025-09-24

**Authors:** Meshref Alshehri, Lyndsay O’Hara, Lauren Leigh Smith, Jonathan Lapin, J. Kristie Johnson, Jacqueline Bork, Surbhi Leekha

**Affiliations:** 1 University of Maryland; 2 University of Maryland School of Medicine; 3 University of MD; 4 University of Maryland Baltimore

## Abstract

**Background:** Daptomycin-nonsusceptible Enterococcus faecium (DNS E. faecium) is an emerging multidrug-resistant pathogen associated with poor clinical outcomes. Prompted by an increase in DNS E. faecium at our institution in 2023 (Figure 1), we sought to characterize patient characteristics and generate hypotheses about potential risk factors. **Methods:** We conducted a retrospective case series of 75 patients with confirmed DNS E. faecium (MIC ≥6) across 10 hospitals (served by a single Microbiology laboratory) within the University of Maryland Medical System between January 1, 2023, and November 30, 2024. Data collected included demographics (age, gender, race, ethnicity), clinical history (prior Enterococcus or VRE infections, immunosuppression), infection characteristics (site, Healthcare-associated (defined as onset >48 hours from admission) vs. Community-onset), and antibiotic exposure (vancomycin, daptomycin, linezolid, rifaximin) within 90 days. Immunosuppression was defined as corticosteroid use (≥10 mg prednisone for ≥5 days), chemotherapy, transplant, neutropenia (ANC < 1 500), or other immunosuppressive conditions. Patients with repeated infections were included once, for their first episode. **Results:** An increase in the incidence of DNS E. faecium was observed at our healthcare system starting in August 2023 (Figure 1). Of 75 patients, 55% were male, 52% were White, 33% were Black, with 92% identifying as non-Hispanic. The mean age was 65 years. A total of 57% of infections were healthcare-associated, with 27% occurring in ICU settings. Specimen sources included blood (20%), urine (5%), and abdominal (48%). Only 5 cases (7%) were considered colonization, while 10 cases (13%) with bacteremia had an ongoing intra-abdominal process. Prior antibiotic exposure was documented in 84% of cases, including vancomycin (51%), daptomycin (15%, high-dose (8-12 mg/kg daily) in 9% of cases), linezolid (13%), and rifaximin (5%). Immunosuppression was present in 39% of patients, and 72% had underlying gastrointestinal pathology (Table 1). Enterococcus was recovered from a previous culture in 20 cases (27%), with 9 (12%) being VRE. Among DNS E. faecium isolates, 59% were vancomycin-resistant, and 48% of those patients had prior vancomycin exposure within the preceding 90 days. **Conclusion:** Patients with DNS E. faecium frequently exhibit prior antibiotic exposure, immunosuppression, and gastrointestinal pathology. However, significant prior exposure specifically to daptomycin was not found. Approximately 40% of isolates were susceptible to vancomycin; therefore, clinicians need to be alert to the possibility of daptomycin resistance in vancomycin-susceptible E. faecium infections. Further study, including case-control analyses, to identify specific risk factors and clarify the role of prior antibiotics vs transmission in the healthcare setting is needed

**Figure:**
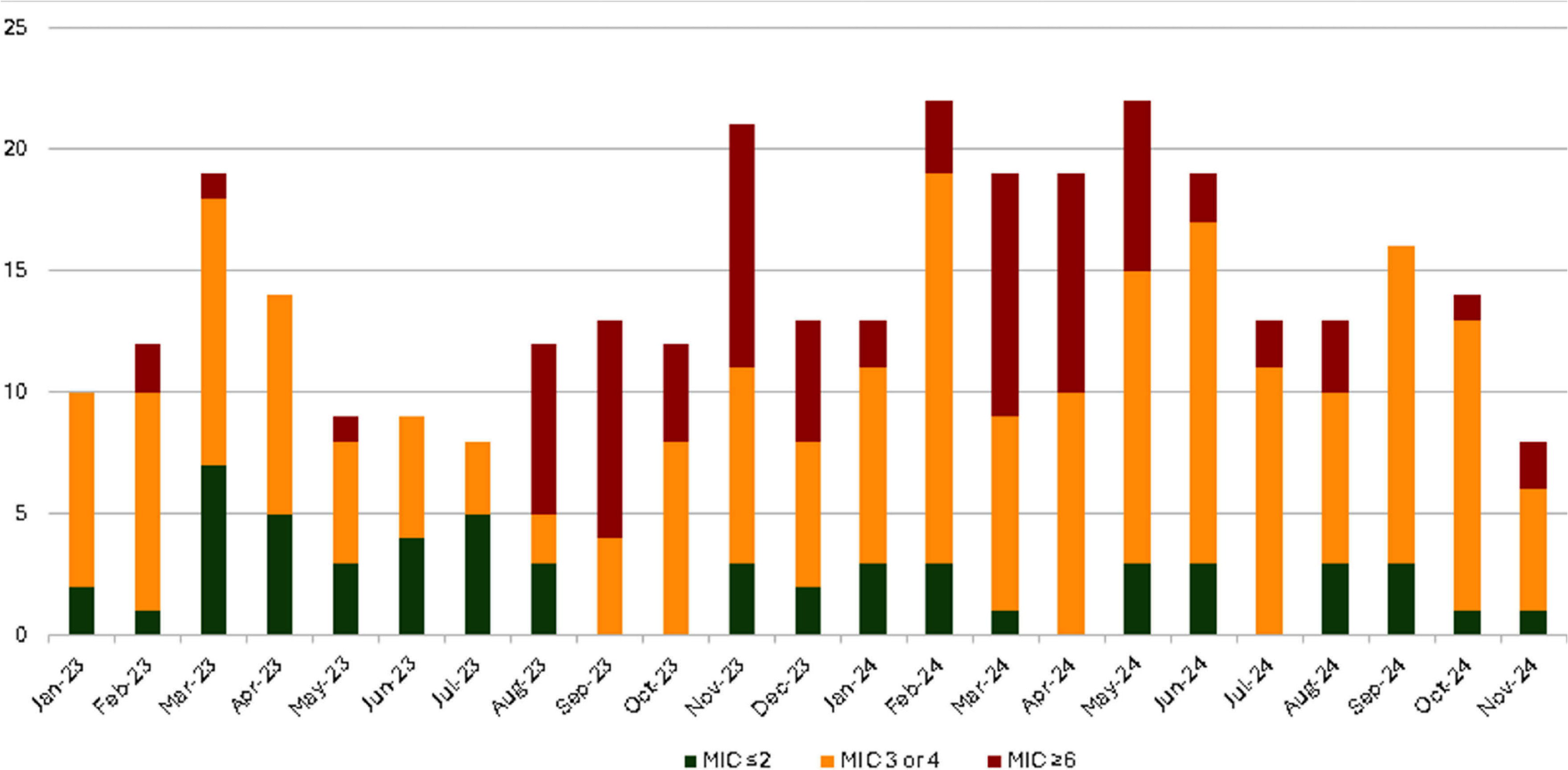

**Figure:**